# Evidence That Phosphorylation of the *α*-Subunit of eIF2 Does Not Essentially Inhibit mRNA Translation in Wheat Germ Cell-Free System

**DOI:** 10.3389/fpls.2020.00936

**Published:** 2020-06-23

**Authors:** Andrey V. Zhigailov, Alena M. Alexandrova, Anna S. Nizkorodova, Gulshan E. Stanbekova, Ruslan V. Kryldakov, Oxana V. Karpova, Nailya S. Polimbetova, Nigel G. Halford, Bulat K. Iskakov

**Affiliations:** ^1^M.A. Aitkhozhin Institute of Molecular Biology and Biochemistry, Science Committee, Ministry of Education and Science, Almaty, Kazakhstan; ^2^Institute of Plant Biology and Biotechnology, Science Committee, Ministry of Education and Science, Almaty, Kazakhstan; ^3^Plant Sciences Department, Rothamsted Research, Harpenden, United Kingdom

**Keywords:** wheat, eIF2, eIF2*α* kinase, GCN2, PKR, eIF2B, mRNA translation, cell-free system

## Abstract

A mechanism based on reversible phosphorylation of the *α*-subunit of eukaryotic initiation factor 2 (eIF2*α*) has been confirmed as an important regulatory pathway for the inhibition of protein synthesis in mammalian and yeast cells, while plants constitute the significant exception. We studied the induction of *Ta*eIF2*α* phosphorylation in germinated wheat (*Triticum aestivum*) embryos subjected to different adverse conditions. Data confirmed that formation of *Ta*eIF2(*α*P) was not a general response, as no phosphorylation was observed under salt, oxidative, or heat stress. Nevertheless, treatment by salicylic acid, UV-light, cold shock and histidinol did induce phosphorylation of *Ta*eIF2*α* of wheat as has been established previously for *At*eIF2*α* in Arabidopsis (*Arabidopsis thaliana*). The influence of *Ta*eIF2*α* phosphorylation on translation of reporter mRNA with different 5′-untranslated regions (5′UTRs) was studied in wheat germ cell-free system (WG-CFS), in which *Ta*eIF2*α* was first phosphorylated either by heterologous recombinant human protein kinase, *Hs*PKR (activated by double-stranded (ds)RNA), or by endogenous protein kinase *Ta*GCN2 (activated by histidinol). Pretreatment of WG-CFS with *Hs*PKR in the presence of dsRNA or with histidinol resulted in intense phosphorylation of *Ta*eIF2*α*; however, the translation levels of all tested mRNAs decreased by only 10–15% and remained relatively high. In addition, factor *Oc*eIF2 from rabbit (*Oryctolagus cuniculus*) bound GDP much more strongly than the homologous factor *Ta*eIF2 from wheat germ. Furthermore, factor *Oc*eIF2B was able to stimulate guanine nucleotide exchange (GDP→GTP) on *Oc*eIF2 but had no effect on a similar exchange on *Ta*eIF2. These results suggest that the mechanism of stress response *via* eIF2*α* phosphorylation is not identical in all eukaryotes, and further research is required to find and study in detail new plant-specific mechanisms that may inhibit overall protein synthesis in plants under stress.

## Introduction

Plants, being immobile organisms, are compelled to withstand various stresses, such as extreme temperatures, nutritional deficiencies, injuries, drought, and salinization, without being able to avoid or weaken them. For these stresses, plants respond by programmed changes in expression of genes at the levels of transcription, processing and translation of mRNA (Floris et al., 2009; Khan et al., 2018). Investigation of translational control mechanisms in plants under stress conditions is of particular importance because numerous changes at the mRNA translation level happen faster than changes at the gene transcription level (Floris et al., 2009; Echevarria-Zomeno et al., 2013). Stresses cause many rearrangements in the protein synthesis apparatus, usually inhibiting translation of most mRNAs: these rearrangements include changes in phosphorylation of translational factors and ribosomal proteins, changes in content and localization of factors and RNA-binding proteins, and formation of stress granules, as well as structural changes of ribosomes (Roy and von Arnim, 2013; Browning and Bailey-Serres, 2015; Merchante et al., 2017).

In eukaryotic cells, stresses are usually accompanied by an inhibition in general protein synthesis to save energy and resources, directing resources instead to the synthesis of special proteins that help the organism to survive the stress (Hinnebusch and Lorsch, 2012). In mammalian cells, one of the main molecular mechanisms for the inhibition of translation of cellular mRNA during stress is the phosphorylation of meIF2 *α*-subunit at Ser51. This is brought about by the action of one or more eIF2*α* kinases (mGCN2, mPKR, mPERK, and mHRI; see *Abbreviations*) and results in a rapid and profound suppression of protein synthesis (Merrick and Pavitt, 2018; Wek, 2018).

Eukaryotic factor eIF2 is necessary for the translation initiation of almost all mRNAs. It consists of three non-identical subunits [α*β*γ] and forms a ternary complex (TC) with GTP and initiator methionyl-tRNA [GTP*eIF2*Met-tRNA_i_^Met^], which then binds to the 40S ribosomal subunit (Hinnebusch and Lorsch, 2012; Wek, 2018). After each round of initiation, factor eIF2 dissociates as an inactive complex [GDP*eIF2*eIF5], and for participation in a new round, the GDP molecule should be replaced with GTP. In mammalian and yeast cells, the affinity of eIF2 for GDP is of two orders of magnitude higher than for GTP (Walton and Gill, 1975); therefore, replacement cannot proceed without the assistance of another important factor, eIF2B, the binding of which first helps to dissociate eIF5 and then to exchange the guanine nucleotides on eIF2 (Hinnebusch and Lorsch, 2012; Merrick and Pavitt, 2018; Wek, 2018). Factor eIF2B forms a complex with [GDP*eIF2] from which GDP may dissociate much more easily because the dissociation constants of complex [eIF2*eIF2B] with GDP or with GTP become almost equal. Thus, the process shifts to TC formation and, therefore, translation initiation proceeds effectively (Bogorad et al., 2018).

Phosphorylation of the *α*-subunit in meIF2 inhibits GDP→GTP exchange catalyzed by meIF2B. The affinity of the exchange factor meIF2B for phosphorylated meIF2(*α*P) is much higher than for the unphosphorylated meIF2*α*. In mammalian cells, the amount of meIF2 is 3–5 times higher than meIF2B; therefore, phosphorylation of only 20–30% of meIF2 leads to the capture of all the available meIF2B molecules, thus preventing new TC formation even with unphosphorylated meIF2. Thus, phosphorylation of eIF2*α* results in rapid inhibition of translation initiation and the shutdown of overall protein synthesis in mammalian and yeast cells (Walton and Gill, 1975; Hinnebusch and Lorsch, 2012; Bogorad et al., 2018; Merrick and Pavitt, 2018; Wek, 2018). A similar mechanism was considered to function as a basic regulatory mechanism in plant cells (Langland et al., 1996; Lageix et al., 2008).

Previously, we have established that the affinity of wheat peIF2 for GDP is only 10 times higher than for GTP (Shaikhin et al., 1992). Consequently, for cyclical functioning of peIF2 in plants there is no need for an eIF2B-like factor, which is strictly required in mammalian and yeast cells. These data suggest that at a sufficiently high ratio of [GTP]/[GDP] concentrations in plant cells, the GDP→GTP exchange on peIF2 can proceed irrespective of its phosphorylation state (Shaikhin et al., 1992). Later, other research groups supported this viewpoint (Janaki et al., 1995; Krishna et al., 1997; Immanuel et al., 2012).

Consistent with this, neither the biochemical activity nor genes encoding a peIF2B-like factor have been found in plants (Immanuel et al., 2012; Echevarria-Zomeno et al., 2013; Browning and Bailey-Serres, 2015). In comparison with the four protein kinases (mGCN2, mPKR, mPERK, mHRI) that are able to phosphorylate the meIF2 *α*-subunit in mammalian cells, only one peIF2*α* kinase, pGCN2, is present in plants (Zhang et al., 2008), and *gcn2* mutants of Arabidopsis (*Arabidopsis thaliana*) lacking *At*GCN2 activity are quite viable, although they demonstrate higher sensitivity to herbicides that disrupt synthesis of amino acids and purines (Lageix et al., 2008; Zhang et al., 2008). Convincing indication of a reduced regulatory role of peIF2 phosphorylation in plants has also been obtained using a phosphoproteomic approach: A study found no difference in phosphorylation of *At*eIF2 subunits in Arabidopsis plants between night (low protein synthesis level) and day (high protein synthesis) (Boex-Fontvieille et al., 2013). Likewise, no phosphorylation of peIF2*α* was observed under osmotic or oxidative stresses (Lageix et al., 2008), viral infections (Zhang et al., 2008), or heat shock (Gallie et al., 1997; Echevarria-Zomeno et al., 2013). Moreover, under stress conditions that cause misfolding of proteins in the endoplasmic reticulum (ER), mammalian cells phosphorylate eIF2*α* using a specific kinase (PERK) in order to reduce overall translation as part of the unfolded protein response. In contrast, the unfolded protein response in Arabidopsis is not accompanied by eIF2*α* phosphorylation, and no changes in protein synthesis level were observed (Kamauchi et al., 2005). Furthermore, using Arabidopsis mutants it was found recently that, while *At*GCN2 does mediate phosphorylation of *At*eIF2*α*, formation of *At*eIF2(*α*P) is not linked to general protein synthesis arrest (Izquierdo et al., 2018). These results show much lesser regulatory and functional relevance of pGCN2 and peIF2(*α*P) in plants relative to other eukaryotes, at least with respect to general protein synthesis.

Nevertheless, the paradigm of mammals and fungi dictates that such a mechanism should also exist and function in plants under certain stress conditions (Lageix et al., 2008; Sormani et al., 2011; Li et al., 2013; Roy and von Arnim, 2013). So, it was shown that peIF2*α* is phosphorylated by pGCN2 in plants subjected to amino acid (Lageix et al., 2008; Zhang et al., 2008) and purine (Lageix et al., 2008) deprivation, with concomitant essential inhibition of protein synthesis (Lageix et al., 2008). Moreover, *At*GCN2 phosphorylates *At*eIF2*α* under such stresses as UV-radiation, cold shock, wounding, treatment with methyl jasmonate, salicylate, and cadmium salts (Lageix et al., 2008; Sormani et al., 2011). Thus, there is no clear understanding of whether the peIF2*α* phosphorylation pathway operates in plants under all kinds of stresses and to what extent general protein synthesis can be inhibited by this mechanism.

In this work, we studied the induction of *Ta*eIF2*α* phosphorylation in germinated wheat (*T. aestivum*) embryos subjected to different adverse conditions. In addition, the effect of *TaeIF2α* phosphorylation on mRNA translation was studied in a wheat germ cell-free system.

## Materials and Methods

### Isolation, Germination and Treatment of Wheat Embryos

Viable embryos were isolated from wheat grains (*T. aestivum*, var. Kazakhstanskaya 4) by the method of Johnston and Stern (Johnston and Stern, 1957). The embryos were washed with distilled water containing 50 U/ml of penicillin, 50 μg/ml chloramphenicol, 50 μg/ml nystatin, and germinated at 26°C for 18 h on 1% agar with 1% glucose and the same antibiotics. After this, the embryos were divided into equal portions (1 g), which were subjected to different subsequent treatments: 1) 4 h at 26°C (control); 2) 4 h at 26°C in the presence of 250 mM NaCl (imitation of high salt and osmotic stress); 3) 4 h at 26°C in the presence of 1 mM H_2_O_2_ (oxidative stress imitation); 4) 4 h at 26°C with 0.6 mM salicylic acid (SA, wound stress imitation); 5) 110 min at 26°C + 10 min under UV light (in UV cross-linker at 2 mJ/cm^2^) + 2 h at 26°C; 6) 4 h at 4°C (cold shock); 7) 4 h at 37°C (heat shock); 8) 4 h at 26°C in the presence of 2 mM L-histidinol (simulation of amino acid starvation). L-histidinol (HisOH) is a potent inhibitor of protein synthesis in mammalian cells, acting not only as an inhibitor of histidine synthesis but also competitively inhibiting histidyl-tRNA^His^ synthetase (Vaughan and Hansen, 1973). After treatments, all samples were frozen in liquid nitrogen.

### Preparation of Wheat Germ Cytoplasmic Extract, SDS-Polyacrylamide Gel Electrophoresis and Immunoblotting

Frozen embryos were ground to a powder in a mortar, and then 5 ml of grinding buffer was added containing 20 mM Tris (pH 7.6), 100 mM KCI, 2 mM MgCI_2_, 5 mM 2-mercaptoethanol, 250 mM sucrose, 1× Cocktail C (phosphatase inhibitor) containing Cantharidin, (−)-p-Bromotetramisole oxalate and Calyculin A (Santa Cruz), 2.5 mM sodium fluoride, 1 mM sodium orthovanadate, 0.5 mM 3-phosphoglycerate, 1.15 mM sodium molybdate. The crude extract was filtered through nylon and centrifuged at 23,000 × g (rotor SW-55, Beckman Coulter) for 20 min to remove the nuclei, plastids, and mitochondria (Iskakov and Madin, 1994). The resulting supernatant was denoted S23-fraction or wheat germ cytoplasmic extract (WGE).

Proteins were analyzed by SDS-PAGE (12.5%) with 0.1% SDS, according to Laemmli (1970). After electrophoresis, the gels were fixed and stained with PageBlue Protein Staining Solution (Thermo Fisher Scientific) or subjected to semi-dry blotting on CBS-apparatus (Thermo Fisher Scientific) in transfer buffer (102 mM glycine, 25 mM Tris base, 20% (v/v) ethanol) for 1 h at 0.8 mA/cm^2^ and 20 V using 0.22 μm pore NitroBind nitrocellulose membranes (GVS Life Sciences).

### Construction of Plasmid With *HsPKR* Gene; Expression and Isolation of *Hs*PKR and Measurement of Its Activity

Plasmid expressing *Hs*PKR with a carboxy-terminal 6His-tag was constructed by polymerase chain reaction (PCR) amplification of *HsPKR* cDNA gene from pUNO-hPRKR plasmid (InvivoGen) using oligonucleotides hPKR-Nde-FW (5′GGCCCATATGGCTGGTGATCTTTCAGCAGG) and hPKR-Xho-Rev (5′ATTGGATCCTTACTCGAGACATGTGTGTCGTTCATTTTTCT). The PCR product was digested with *Nde*I and *Xho*I and ligated into pET23c vector digested with the same enzymes, resulting in ‘Pet23c-HsPKR-6His' plasmid.

Cells of *Escherichia coli* strain BL21(DE3) transformed with Pet23c-HsPKR-6His plasmid were grown in 100 ml of LB medium containing ampicillin (100 μg/ml) at 30°C to 0.5 optical unit of A_600_. The expression of recombinant protein was induced by 0.5 mM IPTG at 30°C for 4 h. Cells were collected by centrifugation, suspended in His-buffer (50 mM NaH_2_PO_4_, 300 mM NaCl, pH 8.0) containing 10 mM imidazole and then lysed by addition of lysozyme (1 mg/ml) and sonication. The cell debris was removed by centrifugation at 10,000 × g for 20 min at 4°C, and the supernatant was combined with PerfectPro Ni–NTA resin suspension (“5-Prime”, Cat# 2900513), shaken at 4°C for 1 h followed by washing of unbound proteins from the column. The resin was washed twice by His-buffer containing 20 mM imidazole at 4°C. The *Hs*PKR-6His bound to the resin was eluted with His-buffer containing 250 mM imidazole and dialyzed against buffer (20 mM Tris(Ac) pH 7.6, 90 mM K(Ac), 2 mM Mg(Ac)_2_) at 4°C for 12 h to remove the imidazole. After dialysis, the *Hs*PKR-6His was concentrated using 10,000 MWCO HY membrane centrifuge columns (Sartorius) according to the manufacturer's manual. Protein concentration was determined by Bradford protein assay (Bradford, 1976). The content of full-length *Hs*PKR-6His in the final preparation was estimated to be 25% of total protein after densitometric scanning following SDS-PAGE and staining.

Poly(rI–rC)—polyinosinic–polycytidylic acid (Sigma) was used as an activator of *Hs*PKR-6His in final concentrations from 1 ng to 10 µg per ml. Electrophoresis of this poly(rI–rC) in agarose gel revealed that the average length was in the 350–400 nucleotides range, permitting the calculation that 1 μg/ml approximately corresponded to 15 nM concentration.

Activity of *Hs*PKR was measured in 20 μl reaction mixtures that contained 10 mM Tris(HCl) pH 7.6; 1 mM DTT; 50 mM K(Ac); 2 mM Mg(Ac)_2_; 10 μM ATP; 20 ng of purified *Hs*PKR and poly(rI:rC) to a final concentration from 1 ng/ml to 10 μg/ml. WG-extract was added so that the total protein concentration was 2.5 μg/μl. The mixtures were incubated for 30 min at 26°C, and then the proteins were separated by SDS-PAGE and the gels subjected to blotting and development as described.

### Antisera

In order to detect phosphorylated *Ta*eIF2*α*, commercially-available phospho-eIF2*α*(S51) polyclonal antibodies were used (Cell Signaling Technology); these had been raised in a rabbit against human phosphorylated eIF2*α*. This antibody has been used successfully in previous studies for the detection of the phosphorylated form of *At*eIF2*α* in Arabidopis (Lageix et al., 2008; Zhang et al., 2008). The amino acid sequence around the phosphorylation site of eIF2*α* is highly conserved among eukaryotes and is almost completely identical between *Hs*eIF2*α, At*eIF2*α*, and *Ta*eIF2*α*. For immuno-detection of *Ta*eIF2*α*, the blots were first ‘blocked' by washing in a blocking solution containing 5% bovine serum albumin (BSA) in TBST buffer (20 mM Tris-HCl, pH 7.5; 150 mM NaCl; 0.05% (v/v) Tween 20) for 1 h at room temperature with gentle shaking. Phospho-eIF2*α*(S51) polyclonal antibodies were used at 1:1,000 dilution in TBST containing 5% BSA then horseradish peroxidase-coupled donkey-anti-rabbit secondary antibodies (ECL) were used as the secondary antibodies in 1:2,000 dilution in a blocking solution. Finally, blot-membrane was developed by a chemiluminescent substrate for peroxidase (Promega) according to the instruction of manufacturer.

To detect *Hs*PKR-6His, the blots were incubated in blocking buffer (5% skimmed milk powder (Sigma) in TBST) overnight at 4°C. The blocked membrane was then incubated with first antibodies (Penta-His mouse antibodies of “5-Prime”, 1:2,000 dilution in blocking buffer) for 1 h at 25°C. After three successive washings in TBST for 20 min each, the membrane was incubated with anti-mouse HRP-conjugate (“5-Prime”) in a 1:2,000 dilution in blocking buffer for 1 h at 25°C. After washing the membrane twice for 20 min in TBST and washing twice for 10 min in TBS, the blot-membrane was developed by a chemiluminescent substrate for peroxidase (Promega) according to the manufacturer's manual.

### *In Vitro* Transcription and Translation

All mRNAs used for *in vitro* translation were obtained by *in vitro* transcription as described earlier (Akbergenov et al., 2004). Transcription *in vitro* of *Eco*RI-linearized plasmid DNAs was carried out using bacteriophage T7 RNA-polymerase (Thermo Fisher Scientific). After incubation for 2 h at 37°C, RNAs were precipitated in the presence of 3 M LiCl followed by double washing with 70% ethanol.

Translation *in vitro* was carried out in WG-CFS as described previously (Madin et al., 2000) with slight modifications. The standard reaction mixture in 25 µl contained: 20 mM Tris(Ac), pH 7.6; 90 mM K(Ac); 2.5 mM Mg(Ac)_2_; 1.6 mM DTT; 0.1 mM spermine; 1 mM ATP; 0.2 mM GTP; 10 mM phosphocreatine; 0.12 mg/ml creatine kinase; 0.1 mM of amino acids; 35% (v/v) of WG-extract (S23), and 2 µg of *in vitro*-transcribed mRNA. The translation efficiency was determined by fluorometric measuring of the activity of reporter protein *β*-glucuronidase (GUS), which was the translation product, as described (Gallie et al., 1991) with 1 mM 4-methylumbelliferyl-*β*-D-glucuronide (MUG) as the substrate. The amount of the fluorescent product (4-methylumbelliferone, 4-MU) in each assay was measured with a TKO 100 fluorimeter (Hoefer).

### Isolation of Wheat Factor WG-eIF2 and Rabbit Factors RR-eIF2 and RR-eIF2B and Guanine-Nucleotide-Exchange Assays

The WG-eIF2 (*Ta*eIF2) was isolated as described previously (Shaikhin et al., 1992) in a procedure that provides about 1 mg WG-eIF2 of 95% purity from 200 g of wheat embryos. The RR-eIF2 (*Oc*eIF2) and RR-eIF2B (*Oc*eIF2B) were isolated from a rabbit reticulocyte lysate (RRL) according to the procedure described by Oldfield and Proud (1992) with slight modifications. This procedure yields approximately 1 mg each of RR-eIF2 and RR-eIF2B, of 90% and greater than 80% purity, respectively. Both procedures include ion-exchange chromatography on S-Sepharose, Q-Sepharose, Mono Q, and Mono S (GE Life Sciences).

The strength of GDP interaction with WG-eIF2 or RR-eIF2 was determined by measuring the loss of [^3^H]GDP from preformed binary complexes with respective eIF2, in the presence of increasing concentration of GTP (from 1 to 400 μM) (Shaikhin et al., 1992). Incubations (50 μl) to form binary [eIF2*[^3^H]GDP] complexes contained 20 mM Tris/HCl, pH 7.6; 100 mM KCl; 1 mM MgC1_2_; 1 mg/ml BSA, 1 mM DTT, 4 µM [^3^H]GDP (350 Bq/nmole) (NEN), and ~200 µg/ml of the respective eIF2 and were kept at 30°C for 10 min. GTP was added in final concentrations from 1 to 400 μM, and reactions were incubated for 10 more min, after which samples were diluted by 3 ml of ice-cold buffer C (50 mM Tris/HCl pH 7.6; 90 mM KCl; 5 mM MgCl_2_) and were filtered through wet nitrocellulose discs. Filters were washed well with the same buffer, dried, and counted in liquid scintillation counter LS-10012 (Beckman Coulter).

Assay of RR-eIF2B activity was carried out according to the procedure of Oldfield and Proud (1992) in 20 µl incubations containing ~60,000 cpm of the respective [eIF2*[^3^H]GDP]; 20 mM Tris/HCl, pH 7.6; 100 mM KCl; 1 mM MgC1_2_; 1 mM DTT; 10 μM GTP and RR-eIF2B at amounts from 10 to 200 ng/20 μl assay. After 5 min at 30°C, samples were diluted with 3 ml of ice-cold buffer C and filtered through nitrocellulose. Filters were washed, dried, and counted as above.

## Results and Discussion

### *Ta*eIF2*α* Phosphorylation Status in Wheat Embryos Germinated Under Various Stress Conditions

Several research groups have shown that *At*GCN2 phosphorylates *At*eIF2*α* in Arabidopsis plants in response to herbicides that impair biosynthesis of amino acids and purines (Zhang et al., 2008; Lageix et al., 2008), suggesting *At*GCN2 to be a good sensor of amino acid and purine nucleotide deficiency. Moreover, AteIF2*α* has been shown to become phosphorylated after UV irradiation and cold shock, as well as after treatment by cadmium salts and plant defense hormones that mediate responses to wounding and phytopathogens, such as methyl jasmonate and salicylic acid (Lageix et al., 2008; Sormani et al., 2011).

However, *At*eIF2*α* is not phosphorylated when plants are subjected to osmotic stress (250 mM NaCl), oxidative stress (1 mM H_2_O_2_), or heat shock (42°C, 2 h), while the same stresses do induce phosphorylation of *Sc*eIF2*α* in yeast cells (*Saccharomyces cerevisiae*), which, like plants, have only one protein kinase for this activity—*Sc*GCN2 (Gallie et al., 1997; Lageix et al., 2008; Immanuel et al., 2012; Munoz and Castellano, 2012).

**Figure 1:**
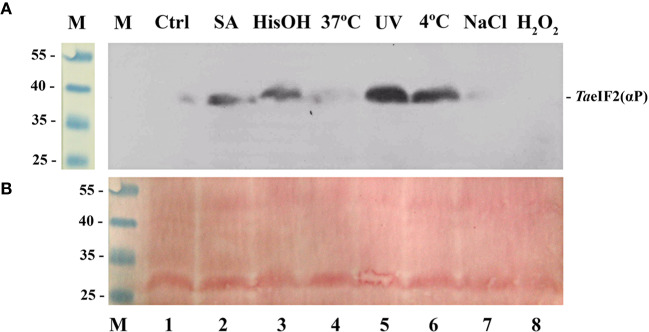
Phosphorylation status of *Ta*eIF2*α* in wheat embryos germinating under normal and stress conditions. **(A)** Fragment of the membrane after immunoblotting using antibodies that specifically recognize the phosphorylated form of eIF2*α*, the position of which is indicated at the right. **(B)** Related fragment of blot-membrane stained with Ponso-S. M: Marker proteins with known molecular mass indicated at the left in kDa. In all variants embryos were first germinated at 26°C for 18 h and then treated as follows: 1) 4 h at 26°C (Ctrl); 2) 4 h at 26°C with 0.6 mM salicylic acid (SA, wound stress imitation); 3) 4 h at 26°C in the presence of 2 mM histidinol (HisOH, simulation of amino acid starvation); 4) 4 h at 37°C (heat shock); 5) 110 min at 26°C + 10 min under UV light (2 mJ/cm^2^) + 2 h at 26°C; 6) 4 h at 4°C (cold shock); 7) 4 h at 26°C in the presence of 250 mM NaCl (imitation of osmotic stress); 8) 4 h at 26°C in the presence of 1 mM H_2_O_2_ (oxidative stress imitation).

In order to investigate this further and to study the patterns of induction of *Ta*eIF2 phosphorylation in wheat, we germinated wheat embryos for 18 h at 26°C and then exposed them to various stresses. Stress conditions were close to those used previously for Arabidopsis plants (Lageix et al., 2008) to allow comparison of the response of monocot and dicot plants. Cytoplasmic extracts (S23) were analyzed by SDS-PAGE and subsequent immunoblotting using commercial antibodies against the phosphorylated form of human eIF2*α* (**Figure 1**).

In accordance with the results previously described for Arabidopsis (Lageix et al., 2008), treatment with salicylic acid (**Figure 1**, track 2), UV-light (track 5), and low temperature (track 6) induced phosphorylation of *Ta*eIF2*α*. To imitate the deficiency of histidine, we treated wheat embryos with L-histidinol (HisOH), which is a potent inhibitor of protein synthesis in mammalian cells. HisOH acts not only as an inhibitor of histidine synthesis but also as a competitive inhibitor of histidyl-tRNA^His^ synthetase (HARS), thereby increasing the level of deacylated tRNA^His^ (Vaughan and Hansen, 1973), which can also bind to the regulatory HARS-like domain of GCN2, resulting in induction of GCN2's kinase activity (Zhang et al., 2003; Wek, 2018). As seen from **Figure 1** (track 3), incubation of wheat embryos with L-histidinol (2 mM, 4 h) caused obvious *Ta*eIF2*α* phosphorylation.

In contrast, almost no phosphorylation of *Ta*eIF2*α* was observed when wheat embryos were subjected to heat shock (**Figure 1**, track 4), osmotic (track 7), and oxidative (track 8) stresses, although these stresses are quite harmful and often encountered by wheat plants. Similar results were earlier obtained with Arabidopsis (Lageix et al., 2008). It is obvious that pGCN2 does not phosphorylate peIF2*α* in response to these kinds of stresses, although heat shock, oxidative and osmotic stresses are known to activate *Sc*GCN2 in yeast cells (Simpson and Ashe, 2012).

Notably, heat shock, high salt, and oxidative stress cause significant inhibition of overall protein synthesis in plants (Echevarria-Zomeno et al., 2013; Merchante et al., 2017), suggesting that other mechanisms (instead of peIF2*α* phosphorylation) may be involved in the global protein synthesis inhibition observed under these conditions (Immanuel et al., 2012; Munoz and Castellano, 2012). Moreover, *gcn2*-mutant plants of Arabidopsis that lack *At*GCN2 activity are phenotypically indistinguishable from wild type plants when grown under normal conditions, although they demonstrate higher sensitivity to herbicides that disrupt the synthesis of amino acids (chlorsulfuron) and purines (8-azaadenine) (Lageix et al., 2008; Zhang et al., 2008). It is noteworthy that some herbicides that do not interfere with amino acid biosynthesis nevertheless induce observable activation of *At*GCN2 (acifluorfen and diuron) and phosphorylation of *At*eIF2*α* (diuron) (Zhang et al., 2008). In the same study, the expression of genes encoding several key enzymes for amino acid biosynthesis was shown for the most part to be independent of *At*GCN2 and not to require phosphorylation of *At*eIF2*α* (Zhang et al., 2008).

These circumstances make the protein kinase *At*GCN2 non-essential for viability of plants and indicate that phosphorylation of peIF2*α* cannot be considered as a universal mechanism for the inhibition of initiation of most mRNA translation in plants that survive multiple stresses. This notion is consistent with many observations that peIF2*α* phosphorylation does not correlate with changes in protein synthesis level *in vivo*. For instance, in a study of Arabidopsis plants grown either in the dark (when protein synthesis level is low) or under daylight (high protein synthesis), no phosphorylation of *At*eIF2*α* was observed at all (Boex-Fontvieille et al., 2013). Another example is that although *At*GCN2 becomes activated under amino acid starvation, resulting in *At*eIF2α phosphorylation, this does not bring about an inhibition of general protein synthesis (Izquierdo et al., 2018).

Thus, there are obvious doubts concerning the role of pGCN2 activation and peIF2*α* phosphorylation in controlling general protein synthesis in plants under stress, and it is evident that formation of eIF2(*α*P) is much less important for plants compared with other eukaryotes (mammals, fungi) (Immanuel et al., 2012; Munoz and Castellano, 2012; Echevarria-Zomeno et al., 2013; Browning and Bailey-Serres, 2015), at least with respect to the general control of protein synthesis.

### Effect of peIF2*α* Phosphorylation on the Rate of Protein Synthesis in a Plant *In Vitro* System

Questions also remain regarding to what extent protein synthesis could be inhibited by phosphorylation of peIF2*α*. Thus, for example, overall protein synthesis has been shown to be strongly inhibited (to 20% level) after treatment of Arabidopsis plants with 8-azaadenine (inhibitor of purine synthesis), a treatment that also induced pronounced *At*eIF2*α* phosphorylation (Lageix et al., 2008). On the other hand, treatment of plants with chlorsulfuron (an inhibitor of the synthesis of some amino acids), which also induced considerable phosphorylation of *At*eIF2*α*, caused only moderate inhibition of protein synthesis, as evidenced by the disappearance of large polyribosomes (over 5-mer) while the amount of smaller polysomes stayed unaffected or even slightly increased (Lageix et al., 2008).

Furthermore, it is long known that addition of the protein kinase, heme-regulated inhibitor (HRI), to rabbit reticulocytes (RR)-lysate results in the phosphorylation of RReIF2*α* and the strong blockage of protein synthesis, while addition of heme can counteract both effects (Farrell et al., 1977). It has been shown that purified RR-HRI does phosphorylate wheat germ (WG)-eIF2*α*; however, the addition of RR-HRI to WG-extract in amounts that were fully inhibitory in the RR-lysate resulted in only a relatively small (25%) reduction of protein synthesis (Benne et al., 1980). Such an inhibition could not be overcome by the addition of purified WG-eIF2 or hemin, and so the authors concluded that even this small inhibitory effect was not associated with any regulatory event, and consequently that there is no heme-related regulatory mechanism in wheat germ (Benne et al., 1980).

In order to study the influence of peIF2*α* phosphorylation on initiation of mRNA translation, we used a wheat germ cell-free system (WG-CFS) in which *Ta*eIF2*α* was first phosphorylated in two different ways: (i) by exogenous recombinant human PKR (*Hs*PKR) that was activated by double stranded (ds)RNA–poly(rI–rC); (ii) by endogenous *Ta*GCN2 activated by L-histidinol (HisOH).

**Figure 2:**
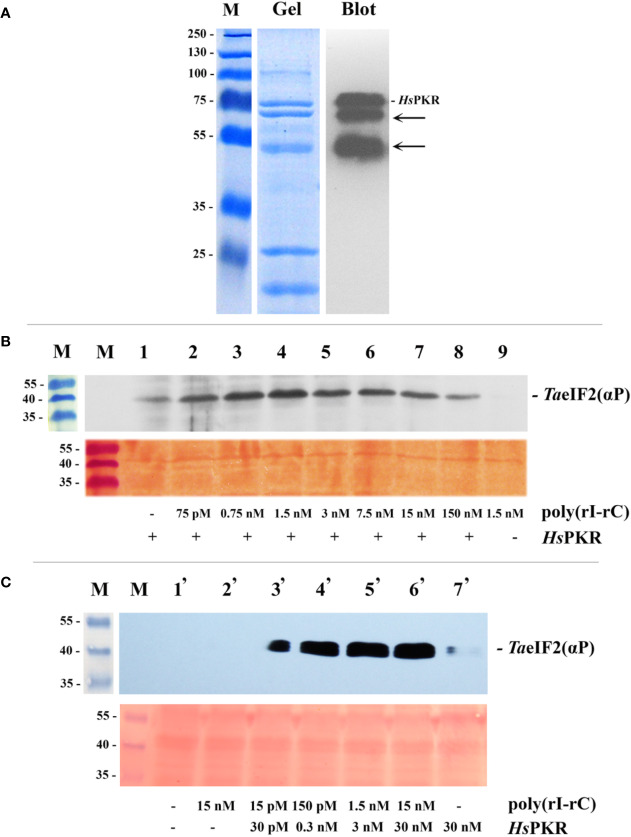
Purification by IMAC of recombinant 6His-tagged *Hs*PKR and testing of its activity in wheat germ extract. **(A)** Coomassie stained SDS-polyacrylamide gel (Gel) and immunoblot (Blot) of purified and dialyzed recombinant *Hs*PKR-6His protein after concentration step and electrophoresis. Position of *Hs*PKR-6His is indicated at the right; the arrows point to degradation products. **(B)** Determination of the activity of the constant amount of recombinant *Hs*PKR-6His induced by increasing concentrations of dsRNA (poly(rI–rC)). Upper panel: The blotted membrane developed using monoclonal anti-phospho-eIF2α(Ser51)-antibodies. Lower panel: Related fragment of blot-membrane stained with Ponso-S. **(C)** Determination of minimal *Hs*PKR-6His concentration required for maximum phosphorylation of *Ta*eIF2*α* during a 20-min reaction. The blotted membrane was developed with monoclonal antibodies against phosphorylated form of eIF2*α*. Position of *Ta*eIF2(αP) is indicated at the right. Upper panel: The blotted membrane developed using monoclonal anti-phospho-eIF2*α*(Ser51)-antibodies. Lower panel: Related fragment of blot-membrane stained with Ponso-S. M: Marker proteins with molecular mass indicated at the left in kDa.

Previously, *Hs*PKR that was partially purified from HeLa cells and activated by poly(rI–rC) has been utilized as a heterologous tool to demonstrate specific phosphorylation of *Ta*eIF2*α* and concomitant inhibition of *Brome mosaic virus* (BMV) RNA translation in WG-CFS (Langland et al., 1996). In our work, we used recombinant 6His-tagged *Hs*PKR that was synthesized in and isolated from bacterial cells using immobilized metal ion affinity chromatography, IMAC (**Figure 2A**). The purified preparation of *Hs*PKR (~64 kDa), which was tagged by C-terminal 6His, contained a substantial amount of a degradation product (~59 kDa) that was truncated from the N-terminus (hence still recognized by Penta-His-antibodies). To verify its functional activity, a constant amount of recombinant *Hs*PKR-6His was supplemented with poly(rI–rC) in different concentrations and tested for its ability to phosphorylate *Ta*eIF2 in wheat germ cell-free extract. After separation of the reaction mixtures in SDS-PAGE and subsequent blotting, the nitrocellulose membrane was developed with commercial antibodies that recognize the phosphorylated form of *Hs*eIF2α (**Figure 2B**).

For detectable activation of *Hs*PKR-6His, very low concentrations (approximately 0.005 μg/ml (75 pM) or less) of poly(rI–rC) were sufficient (**Figure 2B**, track 2; **Figure 2C**, track 2). Maximal activity of recombinant *Hs*PKR-6His kinase was achieved at a concentration of 0.1 μg/ml (1.5 nM) poly(rI–rC). Interestingly, the *Hs*PKR-6His could phosphorylate a small amount of *Ta*eIF2*α* even in the absence of exogenously-added poly(rI–rC) (**Figure 2B**, track 1), indicating that the plant extract may contain some endogenous dsRNA. Upon further increasing the concentration of poly(rI–rC), the activity of *Hs*PKR-6His gradually declined to a minimum at 10 µg/ml (150 nM) (**Figure 2B**, tracks 5–8). Such behavior is in accordance with the known mechanism of *Hs*PKR activation, which involves dsRNA-dependent dimerization and autophosphorylation, implying the optimal ratio to be two *Hs*PKR molecules per one dsRNA (Donnelly et al., 2013).

It is noteworthy that no phosphorylation of *Ta*eIF2*α* was observed in wheat germ extract supplemented with 0.1 µg/ml (1.5 nM) poly(rI–rC) without addition of *Hs*PKR-6His (**Figure 2B**, track 9). This result confirms the previously described inability of *Cowpea mosaic virus* (CMV) dsRNA to inhibit translation of different mRNAs in WG-CFS (Reijnders et al., 1975). Furthermore, the replicative dsRNA-intermediates of *Turnip yellow mosaic virus* (TYMV) or *Turnip crinkle virus* (TCV) that infected Arabidopsis were unable to induce phosphorylation of *At*eIF2*α*, implying the absence of plant kinase equivalent to PKR (Zhang et al., 2008). Recently it was shown that repression of viral RNA translation and the dramatic increase in the biogenesis of RNA-processing bodies (PBs) induced by NB-LRR proteins in response to infection of *Nicotiana benthamiana* are not associated with the phosphorylation of *Nb*eIF2*α* (Meteignier et al., 2016). Nevertheless, high concentrations of dsRNA (≥10 μg/ml) but not single-stranded (ss)RNA have been shown to strongly inhibit *in vitro* translation of BMV RNA in WG-CFS (Langland et al., 1996). Taking into consideration that there is no PKR equivalent in plants (Lageix et al., 2008; Zhang et al., 2008), this result most likely can be explained by nonspecific activation of endogenous *Ta*GCN2, resulting in phosphorylation of *Ta*eIF2*α*, while protein synthesis inhibition could be due to parallel activation of RNA-processing bodies, which may prevent BMV RNA from being translated. A similar explanation could be applicable for the recent work (Niehl and Heinlein, 2019) in which leaf discs of Arabidopsis were treated by very high concentration (0.5 μg/μl) of poly(rI–rC) that (unlike the ssRNA) induced obvious phosphorylation of *At*eIF2*α*. Unfortunately, the concomitant changes in protein synthesis were not measured in that study.

Next, we tried to find the *Hs*PKR-6His and poly(rI–rC) concentrations that would be minimal though sufficient to phosphorylate the maximum amount of *Ta*eIF2*α* molecules in WG-CFS reaction mixtures (**Figure 2C**). This was required to minimize the possible deleterious effects of these compounds on the translation of mRNAs. Such concentrations were determined to be 0.3 nM of *Hs*PKR-6His and 0.15 nM of poly(rI–rC) (**Figure 2C**, track 4); these concentrations were enough to phosphorylate close to the maximal amount of *Ta*eIF2*α* and were used in further experiments.

Note that it was not possible to quantify accurately the proportion of *Ta*eIF2 molecules that were phosphorylated in these experiments. However, it was clear that no detectable phosphorylation of eIF2 occurred in the absence of *Hs*PKR despite the use of highly sensitive antibodies (**Figures 2B, C**). The gradual increase in *Hs*PKR concentration and its activation by dsRNA resulted in strong phosphorylation of *Ta*eIF2 that reached a maximum usually in 20 min (**Figure 2C**). In mammalian systems, as little as 20–25% meIF2 phosphorylation results in 80–90% inhibition of translation (Donnelly et al., 2013).

To exclude nonspecific effects of exogenously-added recombinant *Hs*PKR-6His with possible accompanying contaminants on mRNA translation in WG-CFS, another approach to phosphorylate *Ta*eIF2*α* was taken. As shown in **Figure 1**, L-histidinol (HisOH) at 2 mM concentration was able to activate endogenous *Ta*GCN2 and thus induce *Ta*eIF2*α* phosphorylation in germinating wheat embryos. HisOH is able not only to inhibit the last stage of histidine synthesis but also to competitively inhibit histidyl-tRNA^His^ synthetase (HARS) and thereby increase the level of deacylated tRNA^His^ (Vaughan and Hansen, 1973), which in turn can bind to the HARS-like domain of GCN2, thereby resulting in GCN2 activation (Zhang et al., 2003; Donnelly et al., 2013). The last circumstance makes HisOH a potent (though reversible) inhibitor of protein synthesis, even in mammalian cells, which do not synthesize histidine at all, and thus makes it advantageous for *in vitro* experiments (Hansen et al., 1972).

**Figure 3:**
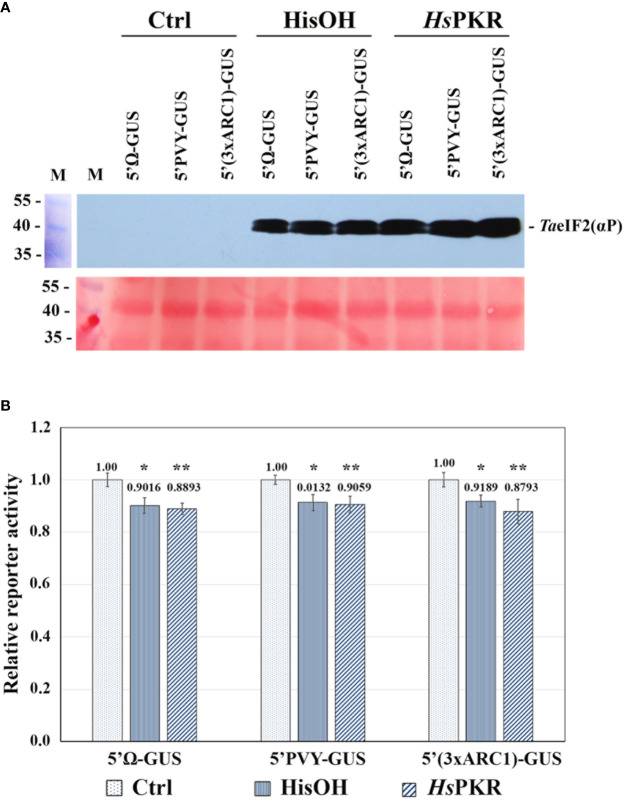
Effects of activated *Hs*PKR-6His and histidinol on *Ta*eIF2*α* phosphorylation and on reporter mRNA translation in WG-CFS. *In vitro* reaction mixtures containing WG extracts were incubated with 2 mM histidinol (HisOH), 0.3 nM recombinant *Hs*PKR-6His and 0.15 nM poly(rI–rC) (*Hs*PKR) or without additions (Ctrl) for 30 min at 26°C followed by addition of mRNAs and further incubation for 1 hour at 26°C, after which GUS activity was measured. **(A)** Testing the phosphorylation status of *Ta*eIF2*α* in reaction mixtures was done as described in **Figure 2**. Presented are the blot-membrane developed using anti-*Hs*eIF2(αP) antibodies (upper panel) and membrane stained with Ponso-S (lower panel). M: Marker proteins. **(B)** Histogram of the levels of different mRNA translation. Reporter activities of different variants were normalized relative to controls that were taken to be 1. GUS activity was measured after 1-h incubation of reaction mixtures at 26°C. Averages ± MD for ten experiments are shown. Asterisks represent significant differences between the control and reactions with addition of histidinol (*) or activated *Hs*PKR-6His (**) (t-test, *P < 0.05, **P < 0.01).

**Figure 3** shows the effects of activated *Hs*PKR-6His and histidinol on phosphorylation of *Ta*eIF2*α* and on the translation in WG-CFS of reporter mRNAs containing various 5′-untranslated regions (5′UTRs). Uncapped *in vitro*-transcribed reporter mRNAs coding for *β*-glucuronidase (GUS) were used, with the following 5′UTRs: 5′UTR of *Tobacco mosaic virus* (TMV) genomic (g) RNA (5′Ω); 5′UTR of *Potato virus Y* gRNA (5′PVY), and artificial translation enhancer 3хARC1 (Akbergenov et al., 2004). The 5′UTR of TMV gRNA is capped in nature though can provide efficient translation in its uncapped form and does not possess an internal ribosome entry site (IRES)-activity. PVY gRNA lacks the cap and has 5′-terminal viral protein genome-linked (VPg) in nature. Its 5′UTR contains an IRES element that can recruit ribosomes internally and provide efficient translation without VPg (Levis and Astier-Manifacier, 1993; Akbergenov et al., 2004). The 5′UTR 3xARC1 contains the artificial translation enhancer that is complementary to the central region (nucleotides 1115-1124) of plant 18S rRNA (Akbergenov et al., 2004).

WG-CFS reaction mixtures were supplemented with either *Hs*PKR-6His (plus poly(rI-rC)) or with histidinol and incubated for 30 min at 26°C, while the reference reactions stayed without such additions. After this incubation, equal aliquots of reactions were analyzed by SDS-PAGE and subsequent western blotting to check the phosphorylation state of *Ta*eIF2*α* (**Figure 3A**). Intensive phosphorylation of *Ta*eIF2*α* occurred in reaction mixtures containing active *Hs*PKR or 2 mM histidinol (HisOH), whereas no phosphorylation occurred without these additions (Ctrl). To study the effect of *Ta*eIF2*α* phosphorylation on protein synthesis level, equal amounts of reporter mRNAs were added to the pre-incubated WG-CFS reactions and incubation was continued further for 60 min. After completion of translation, equal aliquots of the reactions were tested for the *Ta*eIF2 phosphorylation state, which was found to be the same as that demonstrated in **Figure 3A** (data not presented). The efficiency of mRNA translation was quantified by measuring GUS-activity in reaction mixtures using the fluorogenic substrate MUG. The results of translation of uncapped reporter mRNAs containing different 5′UTRs in WG-CFS without and after phosphorylation of *Ta*eIF2*α* are presented in **Figure 3B**. The translation efficiency of all three mRNAs decreased only by 10–15% after pretreatment of WG extract by either active *Hs*PKR or histidinol. In either case, the levels of reporter protein synthesis remained rather high (85–90%), confirming our previously made supposition that protein synthesis in plants may proceed efficiently irrespective of peIF2*α* phosphorylation (Shaikhin et al., 1992).

These results differ from those of Langland et al. (1996), where partially purified *Hs*PKR from HeLa cells, activated by a low concentration (0.1 µg/ml) of poly(rI–rC), completely inhibited translation of BMV RNA in WG-CFS. However, our results agree with the outcomes of *in vitro* experiments done in WG-CFS supplemented by purified rabbit reticulocyte heme-regulated inhibitor (RR-HRI) (Benne et al., 1980; Janaki et al., 1995). While WG-eIF2*α* was obviously phosphorylated by RR-HRI, no significant inhibition of hemoglobin mRNA translation was observed in WG-CFS (Benne et al., 1980; Janaki et al., 1995).

It is noteworthy that we obtained similar results not only with the use of exogenously-added heterologous *Hs*PKR-6His, but also with the endogenous *Ta*GCN2 activated by histidinol. Also, our results agree with recent findings that, while Arabidopsis *At*GCN2, together with *At*GCN1 and *At*GCN20, does mediate phosphorylation of *At*eIF2*α*, formation of *At*eIF2(*α*P) is not accompanied by inhibition of general protein synthesis (Izquierdo et al., 2018).

In comparison with mammalian systems, our results substantially differ. It is known that phosphorylation of meIF2 in the mammalian cell-free system results in strong inhibition of mRNA translation (Benne et al., 1980; Sarre, 1989). Moreover, the addition of L-histidinol to HeLa cells in 2 mM concentration resulted in 90% inhibition of protein synthesis (Hansen et al., 1972). Likewise, in African green monkey kidney cells (BSC-40), expression of recombinant mPKR resulted in 90–95% inhibition of total protein synthesis (Gil et al., 2000).

It is known that in yeast cells the GCN2 is an essential component of the general amino acid control (GAAC) mechanism (Hinnebusch, 2005). The GCN2 senses an excess of uncharged tRNAs, phosphorylates *Sc*eIF2*α* and thus prevents synthesis of many proteins while stimulating synthesis of transcription factor *Sc*GCN4 that activates many genes, including those encoding enzymes involved in the biosynthesis of most amino acids (Hinnebusch, 2005). The *GCN2*-like gene, *AtGCN2*, as well as some other genes of the GAAC mechanism (*AtGCN1*, *AtGCN20*) is present in Arabidopsis, but the gene of transcription factor GCN4 has not been found in plants (Hey et al., 2010). This circumstance indicates that the general amino acid control mechanism is not conserved in plants or is mediated by unknown transcription factors or other plant-specific pathways (Hey et al., 2010; Pratelli and Pilot, 2014).

Thus, data available in the literature (see *Introduction*), together with our results, suggest that phosphorylation of peIF2*α* in plants cannot be considered as a universal mechanism for inhibition of general protein synthesis. Then, why is pGCN2 conserved in plants and why does it phosphorylate peIF2*α* in response to a lack of amino acids, purines, and some other stresses? One possible explanation may be that pGCN2 serves as a sensor of deficiency of amino acids and nucleotides, though it can be also activated in response to some adverse factors. Phosphorylation of peIF2*α* does not prevent translation of most mRNAs, while it may serve as a signal for other mechanisms, which do inhibit general protein synthesis. If so, then the finding and investigation of such mechanisms become an important task.

### Influence of RR-eIF2B on the Exchange of Guanine Nucleotides on RR- or WG-eIF2

As mentioned above, we first established the affinity of plant factor peIF2 for GDP to be only 10 times higher than that for GTP, while for mammalian factor meIF2 the difference in affinities is of two orders of magnitude (Shaikhin et al., 1992). Consequently, at sufficiently high ratios of GTP to GDP concentrations, the exchange of guanine nucleotides on plant peIF2 may proceed without an eIF2B-like factor, whereas eIF2B is strictly required for the exchange in mammalian and yeast cells. Indeed, up to now the biochemical activity of a peIF2B-like factor has not been found in or purified from any plant tissue (Immanuel et al., 2012; Echevarria-Zomeno et al., 2013; Browning and Bailey-Serres, 2015). Nevertheless, there are some genes in the Arabidopsis genome with sequence similarity to genes of mammalian eIF2B subunits, and these have been proposed to encode equivalent proteins in plants (Browning and Bailey-Serres, 2015). On the other hand, there are biochemical data showing that purified WG-eIF2 can exchange guanine nucleotides *in vitro* without the addition of any eIF2B-like protein factor (Krishna et al., 1997).

**Figure 4:**
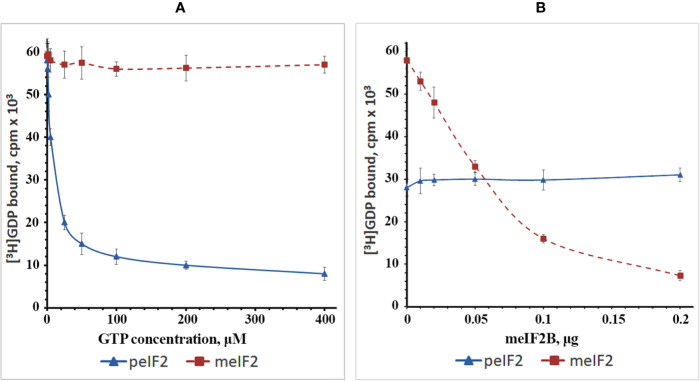
Plots showing the exchange of guanine nucleotides on the purified factors WG-eIF2 (peIF2) and RR-eIF2 (meIF2). **(A)** Replacement of GDP by GTP at different concentrations of GTP in the absence of RR-eIF2B. **(B)** Influence of RR-eIF2B on replacement of GDP by GTP at 10 µM GTP.

To investigate this issue, we isolated WG-eIF2 and RR-eIF2 and studied the kinetics of their interaction with GDP and GTP as well as the influence of RR-eIF2B (**Figure 4**). In the first step, the binary complexes were preformed between [^3^H]GDP and factors WG-eIF2 and RR-eIF2, respectively. Then these binary complexes were titrated by GTP in increasing concentrations (**Figure 4A**). The results show that GTP easily replaced [^3^H]GDP from complexes with WG-eIF2, with GTP at 10 μM replacing about 50% of [^3^H]GDP, at 50 μM about 75%, and at 200 μM about 85%. In contrast, the replacement of [^3^H]GDP on RR-eIF2 did not occur at all at GTP concentrations up to 400 μM.

Next, we tested how RR-eIF2B influenced the exchange of guanine nucleotides on both RR-eIF2 and WG-eIF2 (**Figure 4B**). For this purpose, the binary complexes of [^3^H]GDP with factors RR-eIF2 or WG-eIF2 were supplemented with 10 μM GTP (the concentration at which 50% replacement occurred on WG-eIF2), and then the partially purified RR-eIF2B was added in increasing amounts. As expected, RR-eIF2B greatly increased the replacement of [^3^H]GDP by GTP at 10 μM on factor RR-eIF2; the exchange that was impossible without the addition of RR-eIF2B: indeed, 0.2 μg stimulated almost complete exchange (**Figure 4B**). This showed that the fact that RR-eIF2 did not exchange GDP for GTP in the experiment shown in **Figure 4A** was due entirely to a lack of eIF2B and not to something else associated with the WG-CFS. In contrast, the addition of RR-eIF2B to binary complexes of [^3^H]GDP with WG-eIF2 did not influence the exchange of guanine nucleotides at all (**Figure 4B**), illustrating very clearly the contrast between the plant and mammalian eIF2.

The results confirmed our previous statements that due to the close affinity of WG-eIF2 for guanine nucleotides, the GDP → GTP-exchange on this factor may proceed without the participation of any eIF2B-like factor and independently of whether WG-eIF2 is phosphorylated or not (Shaikhin et al., 1992). Our results also coincide with the findings of another group (Krishna et al., 1997) showing that phosphorylated WG-eIF2 was unable to interact with RR-eIF2B to form a 15S complex similar to the one that forms between phosphorylated RR-eIF2(*α*P) and RR-eIF2B. The authors of that study concluded that the ability of WG-eIF2 to exchange guanine nucleotides independently of any eIF2B-like protein and the inability of phosphorylated WG-eIF2 to interact with RR-eIF2B indicated that WG-eIF2 differs from RR-eIF2 by amino acid sequence and mechanisms regulating its activity (Krishna et al., 1997). These results taken together cast doubt on the existence of an eIF2B-like factor in plants.

### Concluding Overview

Results of the current work show that formation of *Ta*eIF2(*α*P) is not a universal stress response in wheat, in particular under salt, oxidative, and heat stress. Nevertheless, we confirm also that treatment by salicylic acid (stress and defense hormone), histidinol (simulation of amino acid starvation), cold shock, and UV light does induce phosphorylation of *Ta*eIF2*α* in wheat in a similar way as *At*eIF2*α* of Arabidopsis (Lageix et al., 2008).

Using a wheat germ cell-free system, in which factor *Ta*eIF2*α* was pre-phosphorylated either by heterologous mPKR activated by dsRNA, or by endogenous *Ta*GCN2 activated by histidinol, we have shown that the translation level of reporter mRNAs with different 5′UTRs decreased only by 10–15% and remained relatively high. This result is quite different from that obtained in mammalian systems (Hansen et al., 1972; Gil et al., 2000; Immanuel et al., 2012). Additionally, we have shown that factor *Oc*eIF2 from rabbit reticulocytes has a much higher affinity for GDP than the plant homolog, *Ta*eIF2, from wheat germ. Although factor *Oc*eIF2B stimulated the GDP → GTP exchange on *Oc*eIF2, it nevertheless had no effect on the same exchange on wheat germ factor *Ta*eIF2.

Experimental data suggesting the absence of an eIF2B-like factor in plants are very scarce. Shaikhin et al. (1992) first postulated that there was no requirement for an eIF2B-like factor, and Krishna et al. (1997) subsequently obtained experimental evidence in support of this notion. Nevertheless, the issue has remained controversial despite the fact that no evidence of an eIF2B activity has been demonstrated in plants (Browning and Bailey-Serres, 2015). The data reported here show clearly that plant eIF2, unlike its fungal and mammalian counterparts, does not require an eIF2B activity to exchange GDP for GTP and that it is unaffected by mammalian eIF2B.

It should be noted that in addition to the phosphorylation of meIF2*α* there are other mechanisms in mammalian cells that regulate global protein synthesis upon stress conditions and in response to different hormonal signals (Echevarria-Zomeno et al., 2013; Browning and Bailey-Serres, 2015). One of them is the sequestration of factor meIF4E by specific proteins (4E-BPs) upon lack of amino acids or energy and under stress conditions (Hernandez et al., 2010). Phosphorylation of 4E-BPs by an upper-level kinase (mTOR) results in their dissociation, thus permitting meIF4E to interact with m7G-cap and the factor meIF4G, eventually stimulating protein synthesis in response to the availability of amino acids, energy, growth factors, and other stimuli (Hernandez et al., 2010). By contrast, no genes orthologous to mammalian 4E-BPs exist in plants (Hernandez et al., 2010; Echevarria-Zomeno et al., 2013; Browning and Bailey-Serres, 2015).

Recently it was shown that the energy-sensing protein kinase of Arabidopsis (*At*SnRK1) inhibits translation by phosphorylating *At*eIF4E and *At*eIFiso4E at two conserved SnRK1 consensus target sites, which are present in all tested plant factors but absent from homologous mammalian factors (Bruns et al., 2019). The authors considered this mechanism as a novel and direct pathway for translational control in plant cells. However, the universality of such a mechanism requires additional investigation because it was shown earlier that neither *At*eIF4E nor *At*eIFiso4E and *At*eIFiso4G undergo any phosphorylation when plants are subjected to light (high level of protein synthesis) or dark (low level of protein synthesis) (Boex-Fontvieille et al., 2013).

The other mechanism that is known to inhibit global protein synthesis efficiently in mammalian cells is the phosphorylation of threonine 56 (T56) of translation elongation factor 2 (meEF2), catalyzed by specific Ca^2+^/calmodulin-dependent meEF2-kinase in response to starvation, hypoxia, and oxidative stress (Hizli et al., 2013; Sanchez et al., 2019). Phosphorylation of T56 prevents meEF2 from binding to the ribosome, thus strongly inhibiting polypeptide chain elongation.

It was shown earlier that WG-eEF2 can be readily phosphorylated by the RR-eEF2-kinase and this resulted in the inhibition of WG-eEF2 activity in poly(U)-dependent cell-free translation system (Smailov et al., 1993). Thus, the activity of WG-eEF2 can potentially be regulated by phosphorylation. However, the activity of endogenous WG-eEF2-kinase was not detected in wheat germ either *in vitro* or *in vivo*. Furthermore, peEF2-kinase activity was not found in different organs of wheat or other higher plants (both monocots and dicots) (Smailov et al., 1993). Other groups confirmed these results, showing that expression of mammalian eEF2-kinase in wheat germ extract resulted in intense phosphorylation of eEF2 in a Ca^2+^/calmodulin-dependent manner, while there was no such phosphorylation in the absence of cDNA encoding meEF2-kinase (Redpath et al., 1996). Moreover, the translation elongation factors of wheat (both *Ta*eEF1*α* and *Ta*eEF2) were not phosphorylated during seed development, germination, or heat shock (Gallie et al., 1998).

In conclusion, the results obtained in this work together with previous literature data show that plants do not conform to the mammalian and yeast paradigm concerning the regulation of global protein synthesis by reversible phosphorylation of translation initiation and elongation factors. These differences may originate from the autotrophic mode of plant nutrition. Therefore, it is important to look for alternative plant-specific mechanisms that do inhibit protein synthesis in plants under stress conditions (Meteignier et al., 2016). Such alternate mechanisms may also exist in yeast and mammalian cells (Knutsen et al., 2015).

## Data Availability Statement

The raw data supporting the conclusions of this article will be made available by the authors, without undue reservation.

## Author Contributions

AZ prepared recombinant DNAs, HsPKR, WG-CFS, and performed *in vitro* translation reactions and their analyses. AA performed the plant growth, treatments, sample preparation and analyses. AN performed *in vitro* transcription and preparation of mRNA samples. GS and NP prepared rabbit reticulocytes, RReIF2, RReIF2B. OK and AZ prepared TaeIF2 from wheat germ. RK and AZ performed guanine nucleotide exchange reactions and statistical analyses of the data. AZ and BI are the leaders of projects АР05130800 and AP05132532, respectively; BI and NH prepared the manuscript. All authors contributed to the article and approved the submitted version.

## Funding

The work was carried out in the framework of the following scientific grants provided by SC MES RK: AP05132532 to BI; АР05130800 to AZ; AP05131133 to OK; AP05132066 to GS. NH is supported at Rothamsted Research by the Biotechnology and Biological Sciences Research Council (BBSRC) *via* the Designing Future Wheat Programme (BB/P016855/1). BBSRC is part of UK Research and Innovation.

## Conflict of Interest

The authors declare that the research was conducted in the absence of any commercial or financial relationships that could be construed as a potential conflict of interest.

## Abbreviations

eIF2, eukaryotic initiation factor 2; meIF2, mammalian eIF2; peIF2, plant eIF2; WG-eIF2, wheat germ eIF2 = *Ta*eIF2, eIF2 of wheat (*Triticum aestivum*); WGE, wheat germ cytoplasmic extract (= S23); WG-CFS, WG cell-free system; RR, rabbit reticulocyte; RR-eIF2, eIF2 of rabbit reticulocytes = *Oc*eIF2, eIF2 of rabbit (*Oryctolagus cuniculus*); RRL, rabbit reticulocyte lysate; eIF2*α*, the *α*-subunit of eIF2; eIF2(*α*P), eIF2 phosphorylated in the *α*-subunit; eIF2*α*(P), phosphorylated *α*-subunit of eIF2; eIF2*GDP, binary complex of eIF2 and GDP; eIF2B, guanine nucleotide exchange factor (also called GEF); dsRNA, double-stranded RNA; PKR, dsRNA-dependent eIF2*α* kinase; *Hs*PKR, human (*Homo sapiens*) PKR; GCN2, general control nonderepressible 2, kinase of eIF2*α*; HRI, heme-regulated inhibitor, eIF2*α* kinase; PERK, PKR-like endoplasmic reticulum eIF2*α* kinase; (Ac), acetate; BSA, bovine serum albumin; DTT, dithiothreitol; IPTG, isopropyl *β*-D-1-thiogalactopyranoside.
